# MXene/Bacterial Cellulose Hybrid Materials for Sustainable Soft Electronics

**DOI:** 10.3390/ma17225513

**Published:** 2024-11-12

**Authors:** Wojciech Guziewicz, Shreyas Srivatsa, Marcel Zambrzycki, Michał Dziadek, Piotr Szatkowski, Patryk Szymczak, Katarzyna Berent, Marianna Marciszko-Wiąckowska, Marta Radecka, Agata Kołodziejczyk, Tadeusz Uhl

**Affiliations:** 1 Space Technology Centre AGH, AGH University of Krakow, 30-059 Krakow, Poland; 2 Faculty of Materials Science and Ceramics, AGH University of Krakow, 30-059 Krakow, Poland; 3 Department of Materials Engineering, University of British Columbia, Vancouver, BC V6T 1Z4, Canada; 4 Academic Centre for Materials and Nanotechnology, AGH University of Krakow, 30-059 Krakow, Poland

**Keywords:** bacterial cellulose, MXene, soft electronics, viscoelastic properties, piezoresistive sensor

## Abstract

This work evaluated bacterial cellulose (BC) as a possible biodegradable soft electronics substrate in comparison to polyethylene terephthalate (PET), while also focusing on evaluating hybrid MXene/BC material as potential flexible electronic sensor. Material characterization studies revealed that the BC material structure consists of nanofibers with diameters ranging from 70 to 140 nm, stacked layer-by-layer. BC samples produced are sensitive to post-treatment with isopropanol resulting in a change of structural and mechanical properties. The viscoelastic properties of the BC substrates have been studied experimentally in comparison with the PET film. Aged BC substrate showcased similar viscoelastic properties stability, while exhibiting better properties above 70 °C, with total storage modulus change of −15% and loss modulus change of 21%. MXenes prepared using the Minimally Intensive Layer Delamination (MILD) method were screen-printed onto BC substrates and PET films to form MXene/BC (MX/BC) and MXene/PET (MX/PET) devices. The electrical properties results showcased different resistive behavior on both BC and PET substrate samples with different impedance moduli. MX/PET presented lower sheet resistance of around 156 Ω·sq^−1^, while MX/BC was 2733 Ω·sq^−1^. Finally, the MX/BC and MX/PET devices were subjected to repeatable quasi-static load tests and the piezoresistive sensing behavior of the devices has been reported.

## 1. Introduction

Electronics have become an integral part of our lives and although the field may seem to be already well developed, there are countless improvements that can still be made. One of the highly significant topics that has received increasing attention in recent years, driven by increasing environmental awareness, is the biodegradability of commonly used items. The ubiquity of electronic devices in our lives has led to a massive generation of electronic waste, around 53.6 megatones in 2019 [1]. To reduce their impact on the environment, we must explore biodegradable alternatives to printed circuit boards (PCBs) [2] and petroleum-derived polymers. Currently, various biodegradable polymers have been proposed as electronics substrates, such as poly(caprolactone), poly(lactide), poly(lactide-coglycolic acid), cellulose acetate or gelatine [3,4,5].

Another possible solution is the use of bacterial cellulose (BC), which can be produced with the help of bacteria (e.g., Gluconacetobacter xylinus) [6,7] or symbiotic cultures of bacteria and yeast (SCOBY) [8]. The production process does not require specialized equipment or processing methods. The material obtained possesses a complex fibrous structure comprising D-glucose molecules, similar to that of plant-derived cellulose, although it has some variances in its physical and chemical characteristics [9]. In particular, BC exhibits remarkable mechanical strength [10], high water retention capacity (80–90%) and high crystallinity (78–82%) [11]. The fibers produced by bacteria form a complex network with diameters as fine as a few tens of nanometers [12]. BC has been used to manufacture a wide spectrum of materials such as leather alternatives [13,14], films [15], wound dressings [16], sensors [17] or flexible electronics [18]. In addition, efforts are also made to improve its mechanical properties by structurally aligning the fibers [19].

Another class of novel materials that has been gaining increasing attention in recent years is MXenes. They are two-dimensional nanomaterials composed of inorganic compounds, including metal carbides, metal nitrides, or metal carbonitrides [20,21]. The unique feature of MXene compounds lies in the possibility of modifying their chemical structure and stoichiometry, which is achieved through various processing methods, leading to customizable electronic and chemical properties [22]. This distinctive capability serves as a primary motivation for MXene research, distinguishing them from carbon-based nanomaterials, which are restricted by their sp2 carbon structure. The titanium carbide MXene (Ti_3_C_2_T*_x_*-MXene), was first reported in 2011 [23]. During the past decade, Ti_3_C_2_T*_x_*-MXene has risen to prominence among 2D nanomaterials due to its high conductivity (2.4 × 10^5^ S/m) [24], excellent elasticity [25], dynamic response behavior [26], potential for wireless communication [27], solar energy harvesting [28] and microwave absorption performance (92 dB) [29,30]. The two most commonly used methods for MXene synthesis are the hydrofluoric-hydrochloric (HF-HCl) acid etching method and the Minimally Intensive Layer Delamination (MILD) method [31]. While HF-HCl requires the use of highly corrosive and toxic acid, MILD method allows the synthesis of MXenes with the use of HCl and lithium fluoride (LiF), which are more user-friendly to handle. The pure Ti_3_C_2_T*_x_*-MXene films produced by simple vacuum filtration have Young’s modulus of 3.52 GPa [24], while blade-coated MXene results in a freestanding film that has been reported to have a tensile strength of around 570 MPa and Young’s modulus of 20.6 GPa [32]. MXenes have also shown biocompatibility in multiple reported applications [33,34,35]. These advantages offered by Ti_3_C_2_T*_x_*-MXene materials position them as strong candidates for the development of novel materials, particularly when combined with biologically-derived materials such as BC [36].

The paper is organized as follows. The method of material production and processing of BC and MXene nanomaterials is described. The material samples produced of BC and MXene are subjected to material characterization to study the morphology, structure, and chemical composition. The mechanical and electrical properties of BC (untreated, isopropanol treated, and aged sample) are studied and compared with those of commercial flexible PET substrates. Finally, the preparation of MX/BC and MX/PET devices is described with the results of quasistatic tests. The piezoresistive sensing behavior of the MX/BC and MX/PET devices is analyzed and reported with some initial sensor characteristics.

The development of flexible biodegradable electronics is still an unexplored area, driven by advanced materials such as MXene and BC composites [37,38,39]. Using the synergistic properties of these two materials, we can address the demands of next-generation electronic devices that require flexibility, conductivity and environmental compatibility. This article aims to provide initial research results on this topic that authors plan to further develop in the future. An initial evaluation of BC usability in the field of flexible electronics, with respect to its mechanical properties is also considered. The electrical and mechanical properties of the substrate material are crucial for flexible electronics to offer sufficient mechanical support of the whole structure without compromising electrical performance.

The novelty of the presented work focuses on the use of as-prepared MXene precipitate in the form of water-based paste for screen printing in BC, while also evaluating basic characteristics of both MXene and BC. Such an approach allows for relatively simple production of different shapes with pure MXene, while also utilizing a naturally produced, biodegradable polymer.

## 2. Materials and Methods

### 2.1. Bacterial Cellulose Production and Processing

To produce BC sheets, 1000 mL of water was initially boiled and after reaching 100 °C, 10 g of black tea leaves were added for 15 min to brew. After 15 min, the leaves were removed and 60 g of glucose was added and stirred until completely dissolved. The liquid was cooled to 25 °C and then 100 ml of culture medium including SCOBY was added. The sample was then covered and left at 30 °C at ambient humidity and pressure to cultivate. The cultivation time was based on the desired thickness of the sheet. When ready, BC film was taken out of the culture medium and washed with water and isopropanol to remove bacteria and yeasts from the sample. The samples were then left to dry for 24 h on a plastic plate at ambient temperature, pressure, and humidity or taken for further post-processing.

**Figure 1:**
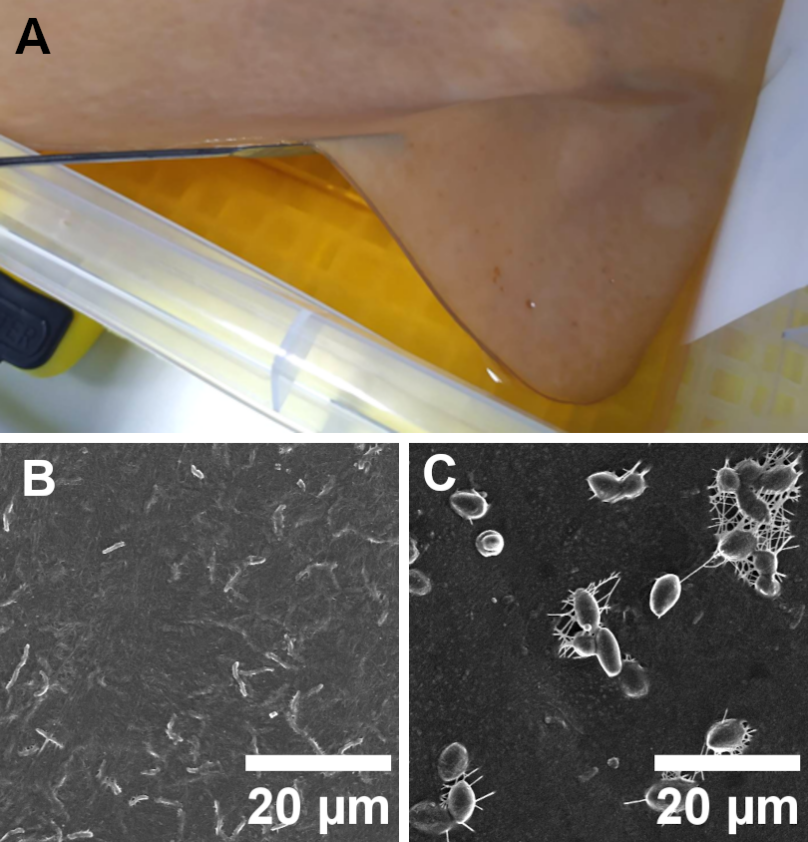
Bacterial cellulose during production (**A**), bacteria (**B**), and yeast (**C**) present in dried, unsterilized material.

One of the post-processing methods involved keeping the sample in isopropanol for 24 h prior to drying to check for any structural changes that could occur. Another method was to age the BC sheet over the 2 month period, leaving it at ambient temperature, pressure and humidity. Figure 1A. Featured BC sheet during production in a culture medium together with the bacteria and yeast present in the material prior to sterilization (Figure 1B and Figure 1C, respectively).

### 2.2. MXene Synthesis

For the synthesis of Ti_3_C_2_T*_x_*-MXene, MILD [40] method with the use of Ti_3_AlC_2_ (<40 μm, Material Research Center, Kiev, UA) as a precursor and HCl (37%, Avantor) and LiF (<100 μm, Sigma-Aldrich, Burlington, MA, USA) was utilized. First, 5 mL of deionized water was added to a polyethylene bottle with a polyethylene-covered magnetic stirrer and heated to 35 °C with stirring at 100 rpm. Then 15 mL of HCl was slowly added and after 5 min of stirring 1.5 g of LiF was added to the mixture. After 5 min of stirring, 1.0 g of Ti_3_AlC_2_ was slowly added over 5 min. The stirring speed was adjusted to 300 rpm and the reaction was carried out for 24 h. After stirring, the obtained etched solution was washed with deionized water and centrifuged at 4500 rpm (for 5 min) multiple times. After each centrifugation process, the clear supernatant was decanted and the washing process was continued until the pH of the solution reached close to 6. Further centrifugation led to the dark green supernatant, indicating the initiation of delamination of the MXene material. Finally, MXene was stored in a container filled with argon.

### 2.3. Morphology, Structure and Chemical Composition Analysis

X-ray diffraction (XRD) was performed with a PANalytical Empyrean diffractometer (Malvern, UK) with CuKα radiation (λ = 1.54056 Å). XRD patterns were recorded in the 2θ angle range from 5° to 90° with a step of 0.026°. Intensities were not normalized in the case of BC results presentation as they were used as received for crystallinity index (CI) estimation. BC CI was calculated using the Segal method with the use of (Equation (1)) [41].

(1) $$\mathrm{CI}=\frac{I_{002}-I_{\min}}{I_{002}}$$

where:

CI—crystalline index [%]
$I_{002}$—intensity of the (002) peak at 22.6°
$I_{\min}$—intensity of diffraction function minimum between 16.8° and 22.6°

Coherent domain size and d-spacing was calculated for MAX, MXene and BC using the Scherrer equation (Equation (2)) and Bragg’s law (Equation (3)):

(2) $$\tau =\frac{K\lambda }{\beta cos\theta }$$

(3) $$d=\frac{n\lambda }{2sin\theta }$$

where:

τ—coherent domain size [nm]
K—shape factor (0.9)
λ—X-ray wavelength [nm]
β—FWHM of reflection measured in 2θ
θ—Bragg angle [°]
d—d-spacing [nm]
n—diffraction order (0.9)

Fourier transform infrared (FT-IR) spectroscopy was performed with a Nicolet iS5 spectrometer by Thermo Fisher Scientific (Waltham, MA, USA). The morphology was examined by atomic force microscopy (AFM) with a Multimode 6 microscope by Bruker (Billerica, MA, USA). AFM measurements were performed in PeakForce Tapping mode with the ScanAsyst-Air probe. Microstructure observations of the surface and cross section were performed using Versa 3D scanning electron microscope by FEI Instruments (Hillsboro, OR, USA). In case of cross-sectional images, the samples were kept in liquid nitrogen and broken. Measurements were made in high vacuum mode with an accelerating voltage of 5 kV, without previous sample sputtering. Fiber diameter (n = 300) and layer thickness (n = 20) measurements were performed on SEM images with ImageJ version 1.54d software using higher contrast settings.

### 2.4. Mechanical Properties

Tensile properties, including Young’s modulus (E*_t_*), tensile strength (*σ_M_*) and break elongation (*ϵ_T_*) of substrate materials, were determined using an Inspekt 5 Table Blue universal tensile machine by Hegewald & Peschke (Nossen, DE, USA) equipped with a 10 N force sensor. The sample length was 50 mm, the sample width was 5 mm, the preload force was 0.1 N, and the test speed was 10 mm/min. Mechanical parameters were calculated by averaging 10 measurements and expressed as mean ± standard deviation (SD). The results obtained were analyzed by one-way analysis of variance (ANOVA) with Duncan post hoc tests, which were performed with Statistica, version 13 by TIBCO Software Inc. (Palo Alto, CA, USA). The results were considered statistically significant when *p* < 0.05.

### 2.5. Viscoelastic Properties

Dynamic mechanical analysis (DMA) was performed with DMA 850 Discovery by TA Instruments (New Castle, DE, USA). The sample was mounted on the device while the middle part of the sample was subjected to oscillating motion with a frequency of 10 Hz and an amplitude of 10 μm. Firstly, the sample was cooled to −60 °C and then heated at a speed of 5 °C/min to a temperature of 80 °C.

### 2.6. Electrical Properties

The electrical conductivity and resistance of the sheets of the films obtained were measured using the four-point probe method using the T2001A3 four-point probe system by Ossila (Sheffield, UK). The I–V curves were collected for each sample in the range up to 10 V, and after reaching the target current, a series of 25 separate resistance measurements were taken and averaged. The sheet resistance and electrical conductivity were calculated according to (Equations (4)) and (Equation (5)), respectively.

(4) $$R_{s}=\frac{C\pi }{ln2}\cdot \frac{\Delta V}{I}$$

(5) $$\sigma =\frac{l}{R\cdot A}$$

where:

R*_s_*—sheet resistance [Ω/cm]
C—geometrical correction factor;
ΔV—voltage drop [V]
I—applied current [A]
σ— electrical conductivity [S]
l—length of sample [cm]
R—measured resistance [Ω]
A—cross-sectional surface area [cm^2^]

The impedance measurements were performed using a Solartron 1260 response frequency analyzer with a 1296 dielectric interface by Ametek Solartron Metrology (Bognor Regis, UK). The materials were examined in the frequency range of 103–107 Hz, AC amplitude of 10 mV, and voltage bias of 0 V. Spectra were analyzed in ZView 3.4 software.

### 2.7. Sensitivity Properties

Sensitivity measurements were performed using a combination of a previously described tensile machine and a 34461A digital multimeter by Keysight Technologies (Santa Rosa, CA, USA). The test setup with mounted samples is provided in the Supplementary Materials. The samples were mounted in the machine and connected to the multimeter. Then they were subjected to cyclic bending from the original (straight) position to the bent position. The offset of the machine arm movement was 10 mm with a speed of 100 mm/min. Between each bending and straightening cycle, there was a break of 5 s. During that the change of resistance of the samples was measured and presented according to the equation (Equation (6)):

(6) $$\Delta R/R_{0}=\frac{R-R_{0}}{R_{0}}$$

where:

R—measured resistance [Ω]
$R_{0}$—initial resistance of the sensor [Ω]

### 2.8. Flexible MX/BC and MX/PET Device Fabrication

**Figure 2:**
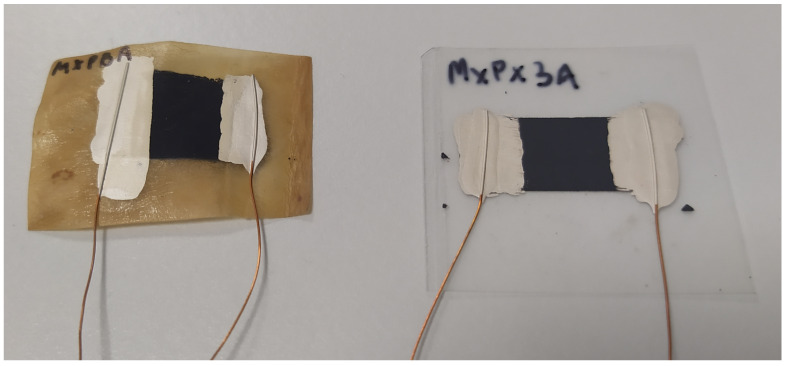
MXene printed onto bacterial cellulose (**left**) and PET (**right**). Silver paste and copper wires were added for electrical and sensitivity measurements.

The screen-printing method was used to produce soft electronic devices. BC and PET sheets were cut into rectangular samples of 20 × 30 mm. MXene paste was used as a water-based paste for screen printing. The screens used had a density of 34T (34 screen fibers per cm^2^). MXene was printed on BC and PET substrate as 10 × 20 mm rectangles (Figure 2).

## 3. Results and Discussion

### 3.1. Structural and Morphological Properties

**Figure 3:**
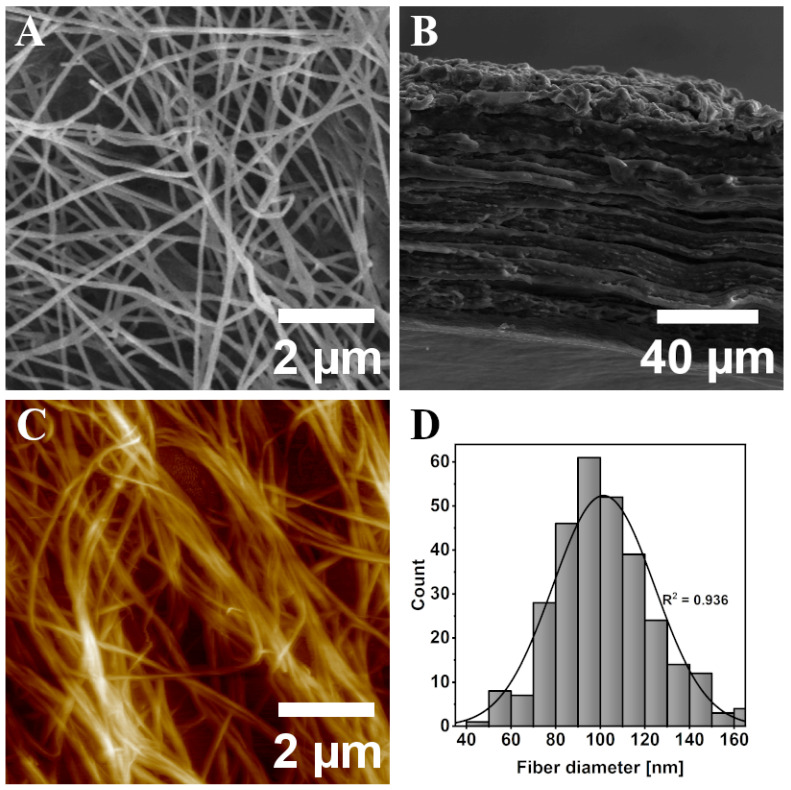
(**A**) SEM image of BC fibers. (**B**) SEM cross-section of dry BC sheet. (**C**) AFM image of BC fibers. (**D**) Fiber diameter distribution chart.

SEM and AFM images are presented in Figure 3. The fibers seen in Figure 3A are long, omnidirectional and have diameters between 70 and 140 nm. In Figure 3B the cross section of the material is presented and it can be seen that the bacteria produce cellulose fibers in an organized, layered manner with a thickness of one layer as low as 700 nm. Such layers form a firm structure and cannot be separated mechanically. AFM topography of the BC surface presented in Figure 3C shows that some of the fibers tend to stick together, forming bigger chunks which may also affect the roughness of the surface. However, no bigger aggregation is observed and that appears to have no real impact during further processing of BC as a screen-printing substrate. The fiber diameter distribution chart is presented in Figure 3D. The regression coefficient of the fitted curve (R^2^ = 0.936) for the diameter of the fibers shows a high correlation with the Gaussian distribution.

**Figure 4:**
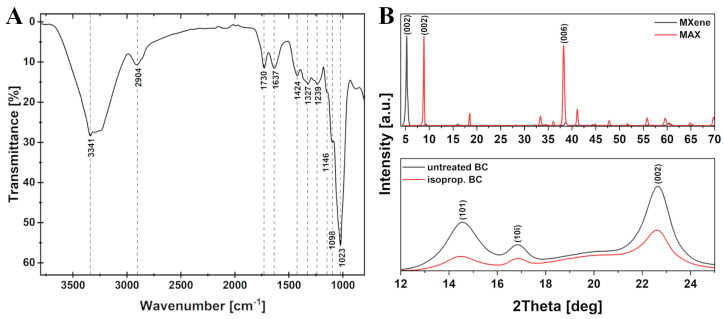
(**A**) FT–IR spectrum of BC. (**B**) XRD diffractogram of MAX precursor and obtained MXene (**top**) and XRD diffractogram of untreated BC and isopropanol treated BC (**bottom**).

FTIR analysis was performed to evaluate the structure and chemical bonds present in the BC. FTIR spectrum (shown in Figure 4A) reveal all characteristic peaks for bonds in cellulose molecules. The signals of stretching of the O-H, C-H, C=O and C=C bonds can be seen at 3341 cm^−1^, 2904 cm^−1^, 1730 cm^−1^, and 1637 cm^−1^, respectively. 1424 cm^−1^, 1327 cm^−1^ and 1239 cm^−1^ peaks appear from the bending of the C-H bond. 1146 cm^−1^, 1098 cm^−1^ and 1023 cm^−1^ peaks can be attributed to the β-1,4-glycosidic bond present between the D-glucose units, whereas all previous peaks come from within the unit. Thus, despite being bacteria-derived, the observed FTIR spectrum is typical of standard cellulose [42].

XRD patterns are shown in Figure 4B. MXene/MAX diffractogram is presented for broader values to show all the lines present. However, the diffraction planes are only attributed to the main peaks because they are used only for the identification of the main parameters of the structure that were presented in Table 1. XRD pattern for MXene/MAX confirms that during the processing stage MAX phase undergoes the etching reaction and forms a 2D MXene structure. That is confirmed by the peak shift of (002) from 9.6° to 5.3° while other diffraction lines disappear due to the formation of the 2D structure. This is attributed to the removal of Al from Ti_3_AlC_2_ and the presence of -OH and -F groups on the surface and in between the planes of MXene formed, which results in increased d_002_-spacing from 0.81 nm to 1.48 nm. In case of BC, the three visible diffraction lines (14.6°, 16.8°, 22.6°) are those of crystalline cellulose and have crystallographic planes attributed to them. D-spacing stays the same for all the planes after isopropanol treatment, however, the coherent domain size decreases significantly. It also affects CI, reducing it from 76% for untreated BC and to 64% for isopropanol treated. In the case of BC, the CI is attributed to the semicrystalline structure of cellulose and the arrangement of the polymer chains inside the fibers. Therefore, its decrease indicates disordering of the polymer chains within the structure of the material. This could be caused by the removal of excessive water molecules from the structure of cellulose polymer that subsequently bonded to the surface -OH groups, which also justifies the reduction of the coherent domain size.

### 3.2. Mechanical Properties

**Figure 5:**
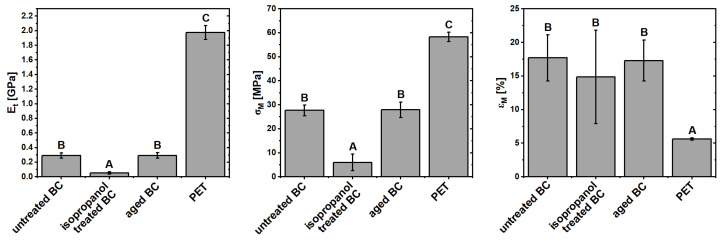
Young’s modulus, tensile strength, and elongation at break for tested samples (n = 10). Same uppercase letters mean no statistically significant difference between the results.

The results of Young’s modulus, tensile strength and elongation at maximum force are presented in Figure 5 and Table 2. Statistically significant differences (*p* < 0.05) between materials are indicated by uppercase letters. Different letters mean that there are statistically significant differences between marked results. The stress-strain curves can be found in the Supplementary Materials. Isopropanol treatment appears to significantly decrease the tensile strength and Young’s modulus of BC material, which correlates with the decrease in CI shown during XRD analysis. PET exhibits a much higher Young’s modulus and tensile strength, however, its elongation at maximum force is much lower (Figure 5). This is attributed to the fact that PET can sustain high elongation to up to 80% due to fiber densification under tensile load, while BC samples break under much lower strains. However mechanical properties for electronics substrate are most important within lower values of elongation, as during stronger stretching the conductive part would most likely disintegrate from the substrate surface due to different flexibility of these two.

### 3.3. Viscoelastic Properties

**Figure 6:**
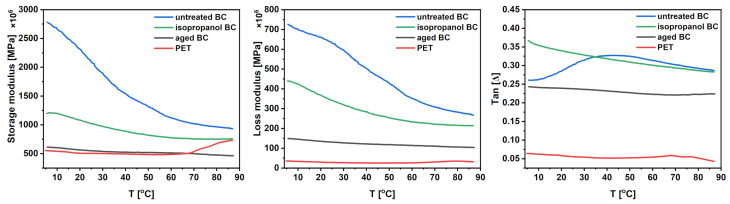
Storage modulus, loss modulus and tangent of samples tested using DMA.

Storage modulus (E′), loss modulus (E″), and their ratio (E″ over E′) were determined using the DMA method, and the results are presented in Figure 6. Measurements of the tested materials indicate a significant difference in their properties. At room temperature, untreated BC exhibits the highest storage modulus (≈2.1 GPa), but when heated with cyclic loading, it decreases significantly (see Table 3). The sample that has been treated with isopropanol shows a lower decrease in storage modulus during the heating cycle, which indicates that its structure is different from that of untreated BC, which is a confirmation of structural analysis. Both isopropanol BC and aged BC have a higher temperature stability during the testing than untreated BC, which is best noticeable in tangent function stability. A very similar situation can be observed in the loss modulus, whereas aged BC and PET indicate a more stable behavior than untreated BC and isopropanol BC. PET has a temperature stability comparable to that of aged BC, however, its possible use at higher temperatures is limited due to the glass transition that occurs around 70 °C which can be noticed in storage modulus measurement. On the other hand, the BC glass transition temperature highly depends on its CI and water content [43], but is above analyzed temperatures. BC is known to be very sensitive to humidity in terms of its mechanical properties, as its different content affects the interactions of intermolecular hydrogen within its structure [44,45]. Recognized differences in viscoelastic properties of untreated BC and aged BC may suggest that BC fibers, as the water particles between them are removed in the drying process and the fibers themselves move closer to each other, are displaced in unfavorable positions afterward. Under cyclic load, the fibers slowly relax, altering their positions, resulting in a decrease of both the storage modulus and the loss modulus, which are attributed to part of the force being stored or lost within the structure of the material. This case is not observed in aged BC, which may be the result of slow self-relaxation of the material over a period of time after drying at ambient temperature and pressure.

### 3.4. Electrical Properties

**Figure 7:**
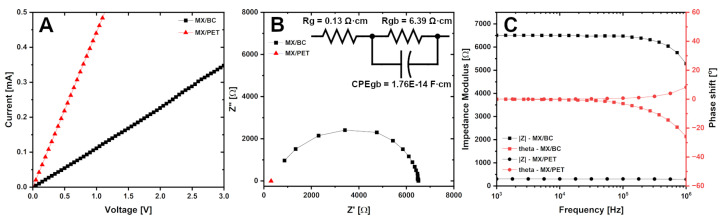
(**A**) Current-voltage response of MXene layers printed on different substrates. (**B**) Nyquist spectra and (**C**) Impedance modulus and phase shift in Bode representation of MXene films on BC and PET substrates.

The DC electrical conductivity of the MXene layers printed on BC and PET substrates was measured using the four-point probe method and the results obtained are presented in summary Table 4. All tested samples demonstrate a linear I–V relationship without notable deviations from Ohm’s law (see Figure 7A). The MXene layers printed on BC are characterized by sheet resistance R*_s_* of 2733 Ω·sq^−1^ (σ = 0.45 S·cm^−1^), while layers printed on PET substrates show resistance of 156 Ω·sq^−1^ (σ = 1.69 S·cm^−1^). The untreated BC substrate exhibits highly resistive behavior and the exact value of resistivity cannot be measured with the equipment used. It should be noted that BC stored in a humid environment (RH ≈ 70%) shows reduced resistance due to molecular water absorption; however, the measured value of sheet resistance is still very high, above 2 × 10^7^ Ω·sq^−1^.

The conductivity measurements were repeated after around 1500 h of storage at ambient temperature and humidity to check how the electrical properties of MXene devices will vary over time. We have found that the sheet resistance of MX/BC and MXene PET samples increases to 201% and 253% of the initial value due to humidity or oxidation of the MXene layer. It should be noted that the MXenes used in the present work are not chemically stabilized with sodium L-ascorbate or citric acid [46]. Using these stabilizers might provide a stable behavior of MXene for a prolonged period. In order to gain further insight into charge transport properties and AC conductivity behavior, MXene layers on BC and PET are studied using impedance spectroscopy and the results are shown in Figure 7B,C. The frequency-dependent response of the MXene layer printed on BC indicates a complex resistive-capacitive behavior, which for granular systems can be described by the following equations (Equations (7) and (8)) [47,48]:

(7) $$Z^{\prime }=\frac{R_{g}}{1+{\left(\omega R_{g}C_{g}\right)}^{2}}+\frac{R_{gb}}{1+{\left(\omega R_{gb}C_{gb}\right)}^{2}}$$

(8) $$Z^{\prime \prime }=\frac{R_{g}}{1+{\left(\omega R_{g}C_{g}\right)}^{2}}+\frac{R_{gb}}{1+{\left(\omega R_{gb}C_{gb}\right)}^{2}}$$

where Z′ and Z″ are real and imaginary parts of the impedance, ω is angular frequency, R*_g_* and C*_g_* are resistance and capacity attributed to the conduction within the grains, while R*_gb_* and C*_gb_* stands for resistance and capacity related to the charge transport between neighboring grains separated by the grain boundaries.

The obtained Nyquist spectrum is characterized by only one developed semicircle (see Figure 7B), which indicates that the contribution of capacitive processes associated with conduction within the MXene grains is too small to be probed at the tested time scales. Therefore, the first terms in Equations (7) and (8) can be replaced by the untreated resistance R*_g_*, while the non-ideal capacitive behavior is modeled by the constant phase element CPE*_gb_* assuming exponent n = 0.9. The equivalent circuit corresponding to the described model is shown in the inset of Figure 7B. The fitting reveals the value of the resistance to conduction in grains R*_g_* = 0.13 Ω·cm, while the contribution to conduction between grains is reflected by the resistance R*_gb_* = 6.39 Ω·cm and the capacitive component C*_gb_* = 1.76 × 10^−14^ F·cm associated with the charge polarization at grain boundaries [47,48]. In turn, the MXene layer printed on the PET substrate demonstrates a much higher conductivity and is characterized by a resistive behavior characterized by nearly untreated resistivity with a small deviation of phase shift appearing in the higher frequency range. The resistance value R*_sum_* is equal to 0.90 Ω·cm, and in this case it can be assumed that this resistance comprises all the resistive and capacitive components of the grain boundary and the grain boundary conduction, while its values are too small to be effectively differentiated from the collected data. These results indicate that the primary reason for the higher resistivity of MXene layers deposited on BC, compared to those of MX/PET samples, is the weaker electrical contact between the separate grains of MXene at BC substrates. In addition, possible interactions between surface -OH groups present in BC and MXene as well as quick water absorption from water-based MXene paste used for screen printing should be considered as influencing factors for future studies.

### 3.5. Sensitivity Properties

**Figure 8:**
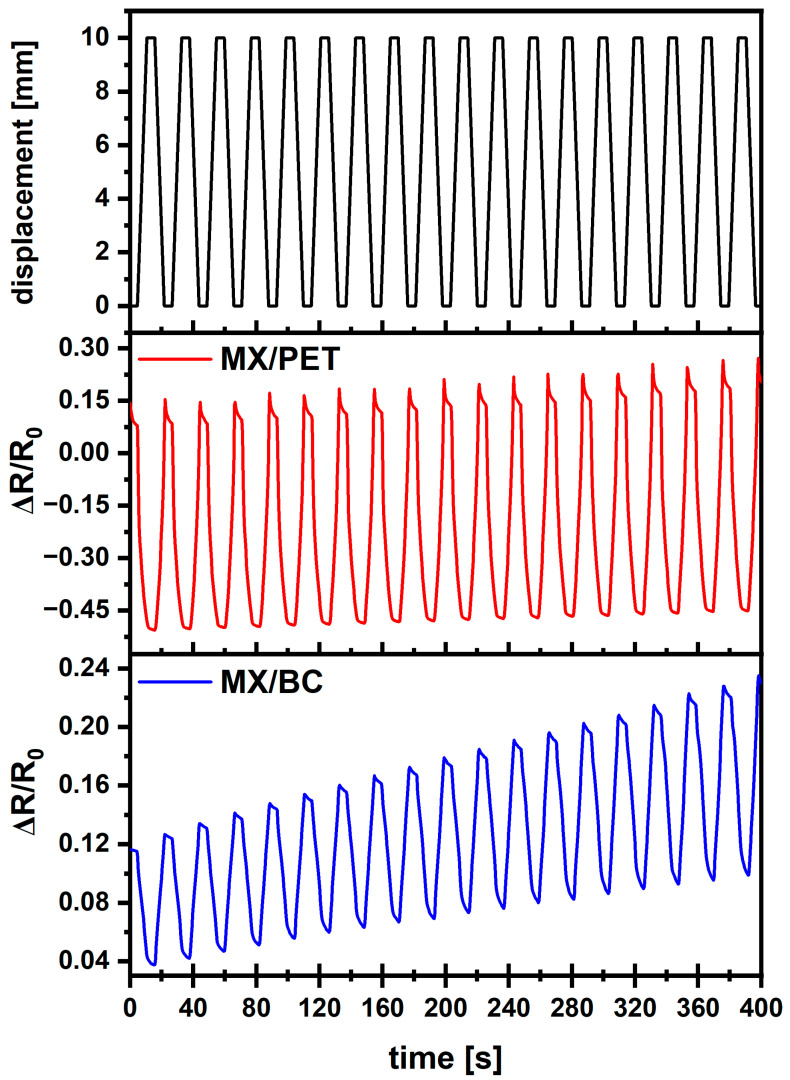
Resistance measurements during cycling loading of MX/PET and MX/BC samples.

Results from the cyclic bending of the sensors are presented in Figure 8. The rise and fall times for MX/PET sensors are 4.3 and 4.6 s, while for MX/BC it is 4.8 and 5.6 s, respectively. Changes in ΔR/R_0_ during the test of MX/PET sample are much greater, around 0.65 per 10 mm, allowing smaller displacements to be detected. At the same time ΔR/R_0_ change for MX/BC is around 0.10 per 10 mm. In addition, the signal is much more qualitatively stable for MX/PET with little variation during all testing cycles compared to that for MX/BC. This difference can be attributed to the fact that PET films are much stiffer and bend in a very repeatable manner, whereas BC tends to have some variations with respect to how it bends with each repetition. MXene films printed onto PET substrates have better sensitive behavior than those printed onto BC substrate. The piezoresistive behavior of MXenes is noticeably better with PET as a substrate, with lower rise and fall time, higher ΔR/R_0_ change and higher signal stability during all cycles. This, along with different electrical behaviors presented in the previous section, results in noticeable differences in sensor performance during initial quasi-static sensitivity tests.

## 4. Conclusions

In this study, the utility of BC as a potential biodegradable substrate for flexible electronics was examined. The fabrication of devices with screen-printed MXene on BC substrate allowed the study of electrical and sensing properties of these devices for future sensing applications. A comprehensive analysis of structural, mechanical and electrical properties was performed for BC in comparison to PET and for MXene. This was done using SEM and AFM imaging, FT-IR, XRD, mechanical testing, DMA, and electrical measurements, while initial tests were also conducted for sensitivity properties.

The results of this paper have shown that BC is produced as a nanofibrous, layered sheet with good mechanical and dielectric properties. Its mechanical properties might change with time or as a result of chemical treatment. The MXene layers deposited showed different electrical behavior in BC and PET. Printing on PET allows for the production of more stable MXene films with regard to their electrical properties and possible sensor production. Because of the more rigid and water repellent surface, MXene films formed onto PET polymer during the screen-printing process may form a more uniform layer that does not interact with the substrate, therefore obtaining better conductivity.

However, interactions between MXene and BC are very complex. Hydroxyl and other surface termination groups present in MXenes may form weak bonds with hydroxyl and oxygen groups present in BC fibers. In addition, high water absorption of BC that is also attributed to its fibrous structure affects the screen-printing process of water-based MXene paste, making it easier to print onto BC than PET, while possibly affecting the structure of the layers. The viscoelastic properties of BC appear to stabilize over time, possibly as a result of the relaxation of the fibrous structure of the material.

Compared to PET, BC offers less reliable mechanical properties, an easier screen printing process of water-based MXene paste, better stability above 70 °C, and most importantly it is naturally produced. However, its interface with MXene seems to decrease electrical properties and overall sensitivity, therefore, it is still requiring more research to reduce these negative effects. Easy and sustainable production makes BC a very interesting and promising material for potential use in flexible electronics, but its post-processing and possible interactions with other materials still need to be thoroughly studied.

## Figures and Tables

**Table 1:** Crystalline index, d-spacing and coherent domain size of investigated samples.

Sample	d-Spacing [nm]	τ [nm]
untreated BC	d_101_ = 0.53 d_10*i*_ = 0.46 d_002_ = 0.35	5.64
isoprop. BC	d_101_ = 0.53 d_10*i*_ = 0.46 d_002_ = 0.35	3.38
MAX	d_002_ = 0.81 d_006_ = 0.20	40.21
MXene	d_002_ = 1.48	29.77

**Table 2:** Young’s modulus, tensile strength and elongation at break for tested samples (n = 10).

Sample	E*_t_* [MPa]	*σ_M_* [MPa]	*ϵ_M_* [%]
untreated BC	289 ± 36	27.6 ± 2.2	17.7 ± 3.4
isopropanol BC	53 ± 17	6.0 ± 3.4	14.9 ± 6.9
aged BC	293 ± 37	27.9 ± 3.2	17.3 ± 3.0
PET	1976 ± 96	58.3 ± 1.9	5.6 ± 0.2

**Table 3:** Storage modulus and loss modulus at ambient and maximum temperature and their percentage loss.

Sample	E′at 25 °C	E′ at 85 °C	E′	E″ at 25 °C	E″ at 85 °C	E″
	**[MPa]**	**[MPa]**	**Change**	**[MPa]**	**[MPa]**	**Change**
untreated BC	2100	950	−55%	630	273	−57%
isopropanol BC	1300	760	−42%	342	214	−37%
aged BC	550	470	−15%	131	105	−21%
PET	505	734	+45%	28	31	+12%

**Table 4:** Summary of the DC conductivity measurements.

Substrate	Thickness [μm]	Sheet Resistance	Conductivity	Resistivity
	**(Substrate + MXene)**	**[Ω·cm^−1^]**	**[S·sq^−1^]**	**[Ω·cm]**
BC (dry)	130 + 0	n.m	n.m	n.m
BC (humid)	130 + 0	2.4 ± 1.1 (×10^7^)	3.2 ± 0.1 (×10^−6^)	3.2 ± 0.1 (×10^6^)
BC (dry)	130 + 10	2733 ± 1321	0.45 ± 0.27	2.73 ± 1.32
PET	70 + 20	156 ± 5	3.21 ± 0.10	0.31 ± 0.01

## Data Availability

The original contributions presented in the study are included in the article/Supplementary Materials, further inquiries can be directed to the corresponding authors.

## Supplementary Materials

The following supporting information can be downloaded at: https://www.mdpi.com/article/10.3390/ma17225513/s1, Figure S1: Stress-strain curves from mechanical tests; Figure S2: SEM images of MX/PET and MX/BC cross-section; Figure S3: Sensivity testing setup using tensile machine and digital multimeter.

## References

[1] Shahabuddin M., Uddin M.N., Chowdhury J.I., Ahmed S.F., Uddin M.N., Mofijur M., Uddin M.A. (2023). A review of the recent development, challenges, and opportunities of electronic waste (e-waste). Int. J. Environ. Sci. Technol..

[2] Zhu J., Wen H., Zhang H., Huang P., Liu L., Hu H. (2023). Recent advances in biodegradable electronics- from fundament to the next-generation multi-functional, medical and environmental device. Sustain. Mater. Technol..

[3] Liu H.J., Chen Z.C., Liang Y.Y., Chang Y.C. (2024). Design of biodegradable gelatin resistive memory with remarkable performance. Org. Electron..

[4] Carrasco-Pena A., Catania F., Haller M., Nippa M., Canterella G., Münzenrieder N. Flexible Thin-Film Temperature Sensors on Gelatin-Based Biodegradable Substrates for the Development of Green Electronics. Proceedings of the 2023 IEEE International Conference on Flexible and Printable Sensors and Systems (FLEPS).

[5] Ko G.J., Kang H., Han W.B., Dutta A., Shin J.W., Jang T.M., Han S., Lim J.H., Eom C.H., Choi S.J. (2024). Materials and Designs for Extremely Efficient Encapsulation of Soft, Biodegradable Electronics. Adv. Funct. Mater..

[6] Nguyen H.T., Saha N., Ngwabebhoh F.A., Zandraa O., Saha T., Saha P. (2021). Kombucha-derived bacterial cellulose from diverse wastes: A prudent leather alternative. Cellulose.

[7] Avcioglu N.H. (2022). Bacterial cellulose: Recent progress in production and industrial applications. World J. Microbiol. Biotechnol..

[8] Laavanya D., Shirkole S., Balasubramanian P. (2021). Current challenges, applications and future perspectives of SCOBY cellulose of Kombucha fermentation. J. Clean. Prod..

[9] Klemm D., Heublein B., Fink H.P., Bohn A. (2005). Cellulose: Fascinating biopolymer and sustainable raw material. Angew. Chem. Int. Ed..

[10] McKenna B.A., Mikkelsen D., Wehr J.B., Gidley M.J., Menzies N.W. (2009). Mechanical and structural properties of native and alkali-treated bacterial cellulose produced by Gluconacetobacter xylinus strain ATCC 53524. Cellulose.

[11] Avcioglu N.H., Birben M., Seyis Bilkay I. (2021). Optimization and physicochemical characterization of enhanced microbial cellulose production with a new Kombucha consortium. Process. Biochem..

[12] Choi S.M., Shin E.J. (2020). The nanofication and functionalization of bacterial cellulose and its applications. Nanomaterials.

[13] Amobonye A., Lalung J., Awasthi M.K., Pillai S. (2023). Fungal mycelium as leather alternative: A sustainable biogenic material for the fashion industry. Sustain. Mater. Technol..

[14] Nguyen H.T., Saha N., Ngwabebhoh F.A., Zandraa O., Saha T., Saha P. (2023). Silane-modified kombucha-derived cellulose/polyurethane/polylactic acid biocomposites for prospective application as leather alternative. Sustain. Mater. Technol..

[15] Feng Y., Zhang X., Shen Y., Yoshino K., Feng W. (2012). A mechanically strong, flexible and conductive film based on bacterial cellulose/graphene nanocomposite. Carbohydr. Polym..

[16] Horue M., Silva J.M., Berti I.R., Brandão L.R., Barud H.d.S., Castro G.R. (2023). Bacterial Cellulose-Based Materials as Dressings for Wound Healing. Pharmaceutics.

[17] Gebrekrstos A., Orasugh J.T., Muzata T.S., Ray S.S. (2022). Cellulose-Based Sustainable Composites: A Review of Systems for Applications in EMI Shielding and Sensors. Macromol. Mater. Eng..

[18] Adamatzky A., Tarabella G., Phillips N., Chiolerio A., D’Angelo P., Nikolaidou A., Sirakoulis G.C. (2023). Kombucha electronics: Electronic circuits on kombucha mats. Sci. Rep..

[19] Wang S., Li T., Chen C., Kong W., Zhu S., Dai J., Diaz A.J., Hitz E., Solares S.D., Li T. (2018). Transparent, Anisotropic Biofilm with Aligned Bacterial Cellulose Nanofibers. Adv. Funct. Mater..

[20] Naguib M., Barsoum M.W., Gogotsi Y. (2021). Ten Years of Progress in the Synthesis and Development of MXenes. Adv. Mater..

[21] Nashim A., Parida K. (2022). A Glimpse on the plethora of applications of prodigious material MXene. Sustain. Mater. Technol..

[22] Li Y., Lai M., Hu M., Zhao S., Liu B., Kai J.J. (2022). Insights into electronic and magnetic properties of MXenes: From a fundamental perspective. Sustain. Mater. Technol..

[23] Naguib M., Kurtoglu M., Presser V., Lu J., Niu J., Heon M., Hultman L., Gogotsi Y., Barsoum M.W. (2011). Two-Dimensional Nanocrystals Produced by Exfoliation of Ti3AlC2. Adv. Mater..

[24] Ling Z., Ren C.E., Zhao M.Q., Yang J., Giammarco J.M., Qiu J., Barsoum M.W., Gogotsi Y. (2014). Flexible and conductive MXene films and nanocomposites with high capacitance. Proc. Natl. Acad. Sci. USA.

[25] Lipatov A., Lu H., Alhabeb M., Anasori B., Gruverman A., Gogotsi Y., Sinitskii A. (2018). Elastic properties of 2D Ti 3 C 2 T x MXene monolayers and bilayers. Sci. Adv..

[26] Srivatsa S., Belthangadi P., Ekambaram S., Pai M., Sen P., Uhl T., Kumar S., Grabowski K., Nayak M.M. (2020). Dynamic response study of Ti 3 C 2 -MXene films to shockwave and impact forces. RSC Adv..

[27] Sarycheva A., Polemi A., Liu Y., Dandekar K., Anasori B., Gogotsi Y. (2018). 2D titanium carbide (MXene) for wireless communication. Sci. Adv..

[28] Raza A., Qumar U., Rafi A.A., Ikram M. (2022). MXene-based nanocomposites for solar energy harvesting. Sustain. Mater. Technol..

[29] Shahzad F., Alhabeb M., Hatter C.B., Anasori B., Hong S.M., Koo C.M., Gogotsi Y. (2016). Electromagnetic interference shielding with 2D transition metal carbides (MXenes). Science.

[30] Ji B., Fan S., Ma X., Hu K., Wang L., Luan C., Deng J., Cheng L., Zhang L. (2020). Electromagnetic shielding behavior of heat-treated Ti3C2TX MXene accompanied by structural and phase changes. Carbon.

[31] Wei Y., Zhang P., Soomro R.A., Zhu Q., Xu B. (2021). Advances in the Synthesis of 2D MXenes. Adv. Mater..

[32] Zhan X., Si C., Zhou J., Sun Z. (2020). MXene and MXene-based composites: Synthesis, properties and environment-related applications. Nanoscale Horizons.

[33] Huang K., Li Z., Lin J., Han G., Huang P. (2018). Two-dimensional transition metal carbides and nitrides (MXenes) for biomedical applications. Chem. Soc. Rev..

[34] Iravani S., Nazarzadeh Zare E., Makvandi P. (2024). Multifunctional MXene-Based Platforms for Soft and Bone Tissue Regeneration and Engineering. ACS Biomater. Sci. Eng..

[35] Wang X., Liang C., Zhang Y. (2024). Functional characteristics and clinical applications of mxene nanoparticles in wound healing. Chin. J. Tissue Eng. Res..

[36] Wu J., Li T., Zhao Q., Wen X., Liu L., Duan J. (2024). Flexible wood-based composite for solar water evaporation and waste heat power generation. Sustain. Mater. Technol..

[37] Huang C., Xiao M., Li Z., Fu Z., Shi R. (2024). Bioinspired breathable biodegradable bioelastomer-based flexible wearable electronics for high-sensitivity human-interactive sensing. Chem. Eng. J..

[38] Zhang W., Ji X.X., Ma M.G. (2023). Emerging MXene/cellulose composites: Design strategies and diverse applications. Chem. Eng. J..

[39] Yamada S. (2024). Biodegradable Mg-Mo2C MXene Air Batteries for Transient Energy Storage. ACS Appl. Mater. Interfaces.

[40] Alhabeb M., Maleski K., Anasori B., Lelyukh P., Clark L., Sin S., Gogotsi Y. (2017). Guidelines for Synthesis and Processing of Two-Dimensional Titanium Carbide (Ti 3 C 2 T x MXene). Chem. Mater..

[41] Segal L., Creely J.J., Martin A.E., Conrad C.M. (1952). Opportunity for new developments in all phases of textile manufacturing. Literature Cited An Empirical Method for Estimating the Degree of Crystallinity of Native Cellulose Using the X-Ray Diffractometer. Technical Report. Apparel Manuf..

[42] Schwanninger M., Rodrigues J., Pereira H., Hinterstoisser B. (2004). Effects of short-time vibratory ball milling on the shape of FT-IR spectra of wood and cellulose. Vib. Spectrosc..

[43] Szcześniak L., Rachocki A., Tritt-Goc J. (2008). Glass transition temperature and thermal decomposition of cellulose powder. Cellulose.

[44] Marcuello C., Foulon L., Chabbert B., Aguié-Béghin V., Molinari M. (2020). Atomic force microscopy reveals how relative humidity impacts the Young’s modulus of lignocellulosic polymers and their adhesion with cellulose nanocrystals at the nanoscale. Int. J. Biol. Macromol..

[45] Cichosz S., Masek A. (2020). IR Study on Cellulose with the Varied Moisture Contents: Insight into the Supramolecular Structure. Materials.

[46] Habib T., Zhao X., Shah S.A., Chen Y., Sun W., An H., Lutkenhaus J.L., Radovic M., Green M.J. (2019). Oxidation stability of Ti3C2Tx MXene nanosheets in solvents and composite films. npj 2D Mater. Appl..

[47] Gutsul O., Szabo O., Kumar N., Pfeifer R., Dzurnak B., Sasitharan K., Slobodyan V., Kromka A., Rezek B. (2023). Electrical properties of MXene thin films prepared from non-aqueous polar aprotic solvents. J. Mater. Res..

[48] Joshi J.H., Kanchan D.K., Joshi M.J., Jethva H.O., Parikh K.D. (2017). Dielectric relaxation, complex impedance and modulus spectroscopic studies of mix phase rod like cobalt sulfide nanoparticles. Mater. Res. Bull..

