# Supplementary material for: MXene/Bacterial Cellulose Hybrid Materials for Sustainable Soft Electronics
Source: Materials (Basel). 2024 Nov 12;17(22):5513. doi: 10.3390/ma17225513 (PMC11595519; doi:10.3390/ma17225513)
Supplement: Supplementary file 1 [file materials-17-05513-s001.zip › materials-3291542-supplementary.pdf]

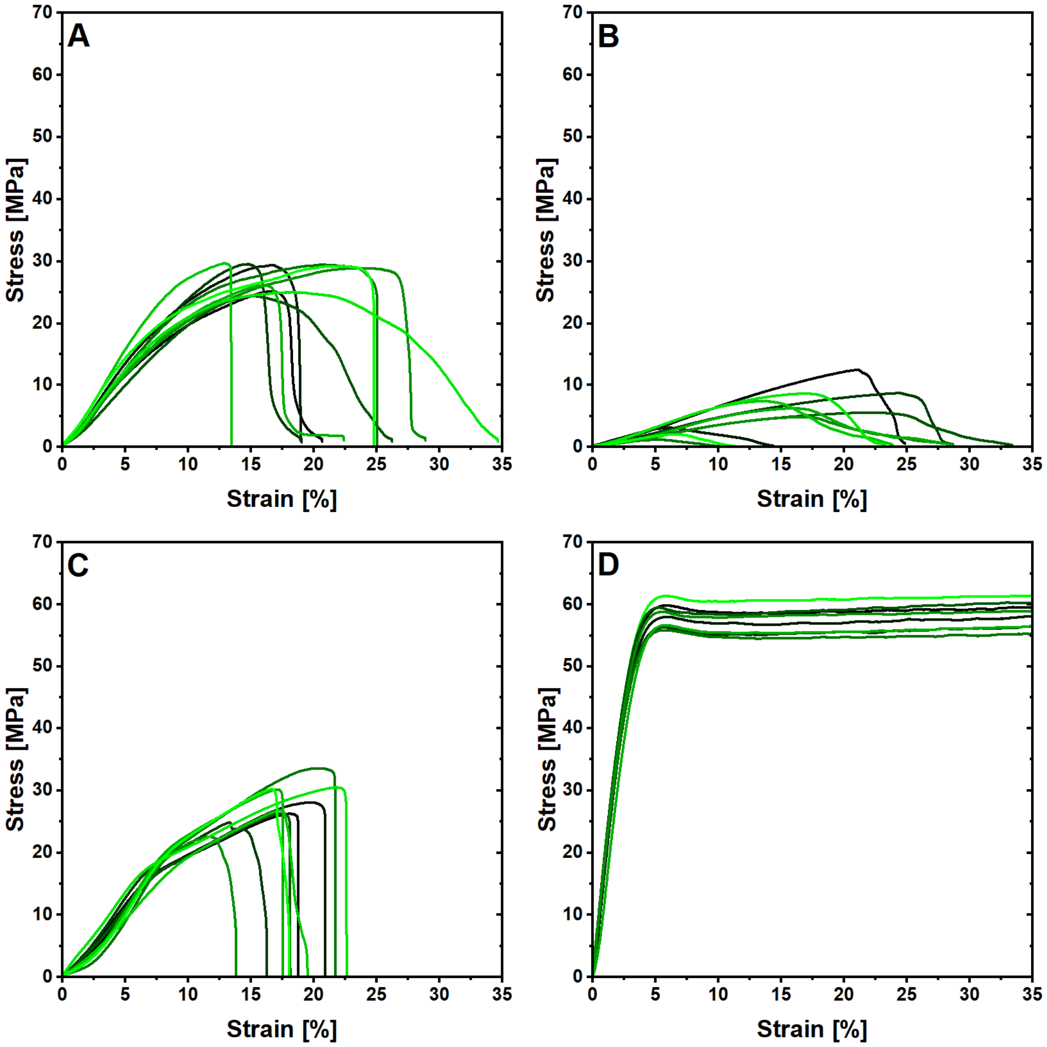

Figure S1. Stress-strain curves from mechanical tests (A – untreated BC, B – isopropanol BC, C – aged BC, D – PET).

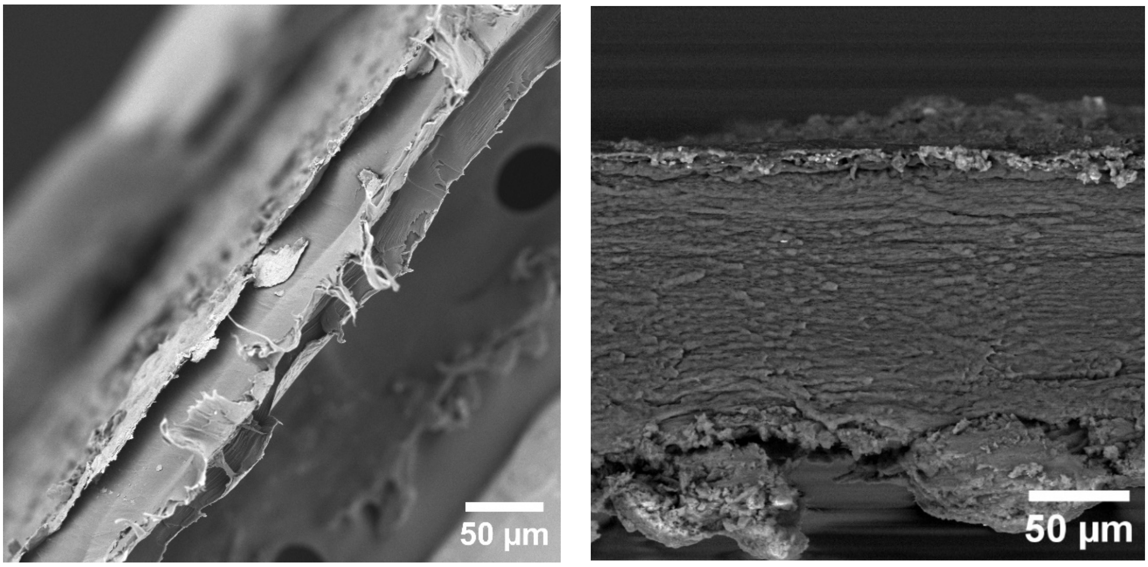

Figure S2. SEM images of MX/PET (left) and MX/BC (right) cross-section.

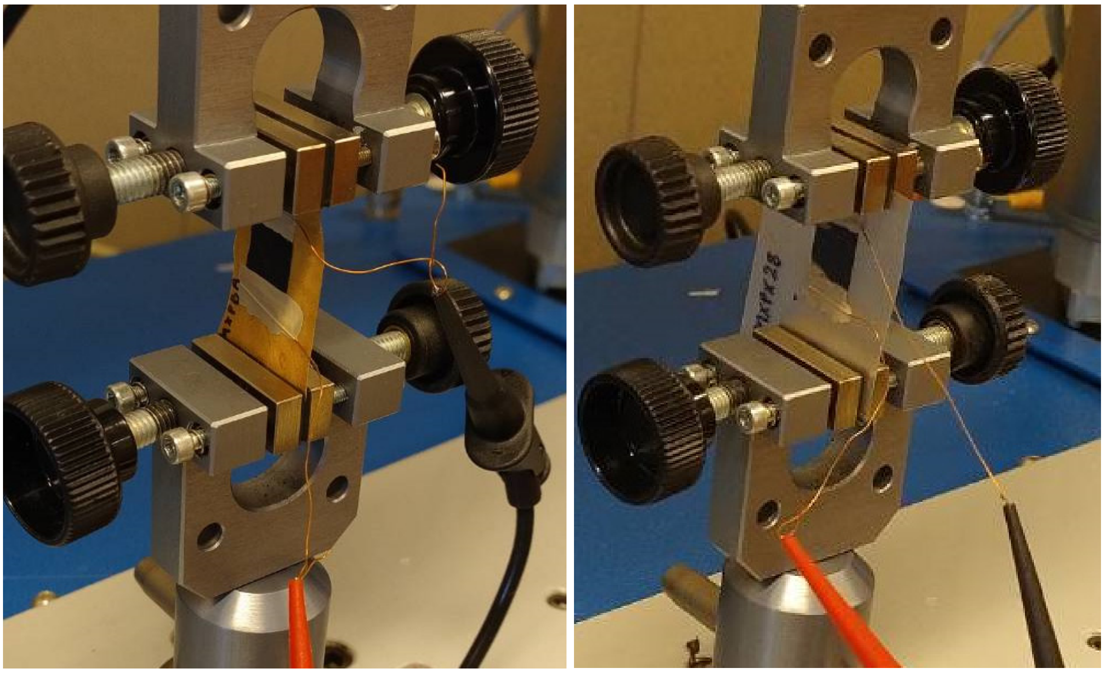

Figure S3. Sensivity testing setup using tensile machine and digital multimeter (left – Mxene/BC, right – Mxene/PET).
